# Iranian regional cancer incidence is misclassified in neighborhood’s provinces 

**Published:** 2016

**Authors:** Mohamad Amin Pourhoseingholi, Ahmadreza Baghestani, Alireza Abadi, Nastaran Hajizadeh

**Affiliations:** 1 *Gastroenterology and Liver Diseases Research Center, Research Institute for Gastroenterology and Liver Diseases, Shahid Beheshti University of Medical Sciences, Tehran, Iran*; 2 *Department of Biostatistics, Shahid Beheshti University of Medical Sciences, Tehran, Iran*

 Cancer is one of the most important causes of death worldwide. There are 14 million new cases of cancer, which recorded globally each year (1). In Iran, cancer is an important health problem (2) and the gastrointestinal cancers are the most frequent cancers among Iranian males and second among females (3, 4). Cancer registration is an important source for measuring the burden of cancer in a population and these data would be useful for policy makers in order to responsible for the provision of health and oncology services, calculating burden of diseases, medical consultations, etc (5). The World Health Assembly passed a resolution in May 2005, recommending all governments to create and implement cancer control plans (6). Iranian ministry of health commenced a National Cancer Registry in 2004, and its data became available by 2006. On that date, in Iran, cancer services were overwhelmed and most patients, who went to a providing care facility, had advanced diseases, with no specific record of diagnosis. Therefore, a Comprehensive National Cancer Control Program (CNCCP) was designed (7) to assess this objective. However, the history of cancer registry foundation was backed in 1955, when the first activities to organizing cancer reporting were initiated by establishing the Cancer Institute at University of Tehran (8). 

In cancer registry, the complete coverage of all cases is the key criterion for data quality (9). Completeness is defined as the proportion of incident cancer cases that is registered (10). The completeness level of cancer registration is one of the main parts of quality control in such registration system (11). In the absence of this completeness, there would be an underestimation for incidence (and also burden) of any cancers. One problem regarding cancer registry is the geographical part that cancer cases diagnosed and the real hometown of the corresponding registered patients. A population-based cancer registry is usually easier to achieve in smaller countries, while in a vast geographical territory, such as Iran, with different climate and cultural diversity, there are technical and logistic problems. A study on the accuracy of gastric cancer incidence in Ardabil province indicated that none of the sources of pathology reports, death certificates and medical records individually or collectively fully cover the incidence of gastric cancer (12). Although legislations in place are indeed helpful in establishing pathology-based registries (8), misclassification in reported new cancer cases among provinces is still a potential problem for achieving a full registry in each territory. Misclassification occurs when new cancer cases are diagnosed and registered in neighborhood provinces (instead of their hometown) due to a low facility in their own provinces, which causes people moving to the neighborhood provinces for their diagnosis or other medical treatments. According to the Iranian annual of national cancer registration report for the year 2008 (13), Razavi Khorasan, North and South Khorasans, (located in the East of Iran) as the neighborhood provinces were presented. Also, Ardabil, as well as East and West Azerbaijan (located in the northwest of Iran) are in neighborhoods. These six provinces were selected to imply the misclassified coverage of registered cancer cases. The reported coverage for Razavi Khorasan exceeds than what was expected, according to the national cancer registration reports (155.5%), whereas the other two provinces with only 34.8% and 41.4% of the expected coverage. There was a same story for East Azerbaijan with 123.6% and its neighbors. Meaning, there were a bunch of diagnosed cases that moved there due to better medical facility, and registered as the resident of where they were diagnosed. 

Although in cancer registry system, the address and telephone number is asked as the extracted information, it is still some patients who are registered in wrong places. A study on cancer registry of Fars province, which investigated the coverage of registered data in southern Iran for almost ten years, revealed that the data was still deficient in recording patients’ phone number and address. In addition, the information for the patients’ national identification number was inadequate (14).

## Conclusion

Accurate cancer incidence data are essential such as planning, monitoring, as well as evaluating national and regional cancer control programs (15). In Iran, there are provinces with higher or lower incidence of GI cancers and policy makers who employ these data to allocate the facilities and resources according to these incidences statistics. Although among medical indexes, incidence is a familiar projection in the assessment of the burden of diseases, in the presence of misclassification, the statistics would be inaccurate and unreliable regionally and make underestimation (or overestimation) for some provinces. Improving the quality of the cancer registry program in Iran will require funding for appropriate infrastructure, enhanced hardware and software resources, and increased expert staffing (14). Besides, better registration with more accuracy regarding the residency of patients, it would be beneficial to improve the estimation of local cancer incidence. In the absence of such revised data, statistical technique could be used to adjust the problem of misclassification (16). 
